# SARS-CoV-2 reinfection: a possible contributing factor to long COVID in children and adolescents

**DOI:** 10.3389/fped.2026.1691052

**Published:** 2026-06-16

**Authors:** Rosela Lucero Chipol-Ceja, Jaime Morales-Romero, Carlos Alonso Rivero-López, María del Sagario Pérez-Callejas, Geovani López-Ortiz, Luis Del Carpio-Orantes, Claudia Iveth Spinoso-Torres, Santiago González-Periañez, María de Jesús Rodríguez-Cordoba, Liliana Ovando-Diego, Jorge Iván Zurutuza-Lorméndez

**Affiliations:** 1 Family Medicine Unit No. 34, Mexican Institute of Social Security, Catemaco, Mexico; 2 Institute of Public Health, Universidad Veracruzana, Xalapa, Mexico; 3 Division of Postgraduate Studies Family Medicine Subdivision, Faculty of Medicine, National Autonomous University of Mexico, Mexico City, Mexico; 4 Academic Secretary, Universidad Veracruzana, Xalapa, Veracruz, Mexico; 5 Servicios de Salud de Veracruz, Xalapa, Mexico; 6 Family Medicine Unit No. 36, Mexican Institute of Social Security, Cardel, Mexico; 7 Faculty of Bioanalysis, Universidad Veracruzana, Xalapa, Mexico; 8 Faculty of Medicine, Universidad Anáhuac Veracruz, Xalapa, Mexico; 9 Family Medicine Unit No. 66, Mexican Institute of Social Security, Xalapa, Mexico

**Keywords:** child health, COVID-19, epidemiological factors, post-acute COVID-19 syndrome, post-infectious disorders, SARS-CoV2

## Abstract

**Background:**

Following the end of the COVID-19 pandemic, attention shifted towards patients who developed sequelae, persistent symptoms, or relapsing or remitting symptoms of new conditions after a prior history of acute SARS-CoV-2 infection. The objective of the present study was to identify the prevalence, clinical characteristics, and potential associated factors of long COVID in children treated during the pandemic in a primary care unit.

**Methods:**

A cross-sectional analytical study was conducted from January to December 2022. Children under 18 years of age and their parents were included in the study if they had been treated at the Mexican Social Security Institute. Two distinct manifestations of long COVID were considered: (a) “persistence”, defined as continuous symptoms beginning in the acute phase and lasting for more than 3 months; and (b) “post-COVID conditions”, defined as new or recurrent symptoms lasting for more than 3 months, appearing after the acute episode, and not associated with any active disease or infectious condition. An exploratory binary logistic regression analysis was performed to identify associated factors, using an odds ratio (OR) as the measure of association.

**Results:**

The study included 349 children and adolescents. The prevalence of long COVID was 11.8% (_95%_CI 7.8%–17.5%). For “persistence”, the most frequent symptoms were cough (50%) and rhinorrhea (15.4%); for “post-COVID conditions”, the most common symptoms were myalgia (33.3%), asthenia and irritability (26.7% each), and constipation (20%). Multivariate analysis revealed that the associated factors for individuals aged over 8 years were a history of reinfection (OR 9.7, _95%_CI 1.6–58) and BMI at the time of the survey (OR 1.1, _95%_CI 1.0–1.2), while for those aged under 8 years, the associated factor was male sex (OR 4.7, _95%_CI 1.3–17.3). It is important to emphasize that these results are the product of an exploratory analysis and aim to create and test new hypotheses.

**Conclusions:**

For healthcare professionals, it is crucial to consider the possibility of long COVID, as this study indicated that approximately 12% of children and adolescents may be affected. Further research is necessary to better understand and manage long COVID in pediatric populations and to investigate the association between reinfections and their increased prevalence.

## 1 Introduction

Following the declaration of the end of the COVID-19 pandemic caused by SARS-CoV-2 on 5 May 2023 by the World Health Organization (WHO), scientific research groups have begun focusing on evaluating the sequelae of the disease, along with the persistence of symptoms or the emergence of new symptoms without an apparent cause in individuals with a prior history of COVID-19 (1, 2). This new chronic manifestation of the disease initially lacked a clear definition or name, but it was identified almost concurrently with the progression of the pandemic, with reports of its emergence dating back to 2020 and attributing its occurrence to disease sequelae and relapses (3–5).

This variability in clinical recognition has complicated diagnosis, resulting in reported prevalences ranging from 2.6% to 93.3%. This range has been attributed to differences in disease severity, age, vaccination status, and a variety of other factors, with an average prevalence of approximately 42.1% in the general population (6). In 2024, the National Academies of Sciences, Engineering, and Medicine (NASEM) in the United States proposed a comprehensive definition of long COVID, integrating multiple perspectives and specifying that: “There must be a diagnosis of COVID-19 prior to the onset of symptoms, regardless of the severity of the acute condition; symptoms must persist for a minimum duration of three months, with onset during the acute phase or later, presenting as continuous, recurrent, remitting, or progressive, and affecting one or more organs" (7) [SIC].

In children, this issue has proven to be significantly more complex due to the higher proportion of asymptomatic cases during the acute phase in this population. Reported prevalence ranges from 1.06% to 70% (8), with approximately 4% considered the general average (9). Those most affected include those who required hospitalization, experienced severe disease, or were unvaccinated (i.e., had not received at least one dose at the time of the acute episode) (8–10). Several studies have described the clinical characteristics and risk factors of long COVID in children, with the most frequent symptoms including fatigue, myalgia, headache, anosmia, dysgeusia, cough, facial paralysis, hoarseness/dysphonia, acute hearing loss, tinnitus, vertigo/dizziness, anxiety, and depression (11–14). This condition represents an economic burden due to the need for continuous and long-term care, in addition to having a significant impact on quality of life (15, 16).

The majority of studies on long COVID in children are based on population cohorts from the United States (10–12, 15–17). However, studies conducted on different cohorts in other countries have identified varying prevalences, clinical characteristics, and risk factors (8, 9, 13, 14), making population-specific research essential. In Mexico, studies have primarily focused on the adult population (18), while those involving pediatric patients have mainly addressed acute disease and vaccination in this age group (19). Evidence regarding pediatric long COVID in primary care settings in Mexico remains scarce. This hinders timely case recognition, increases the risk of underdiagnosis, and limits the development of diagnostic, follow-up, and treatment strategies adapted to the local context and healthcare system.

In this context, the objective of this study was to identify the prevalence, clinical characteristics, and potential associated factors of long COVID in children with symptomatic acute illness who were treated during the pandemic in a primary care unit.

## 2 Materials and methods

### 2.1 Study population

A cross-sectional analytical study was conducted from January to December 2022. The included patients were invited to the waiting area of a Family Medicine unit of the Mexican Social Security Institute, a primary care facility located in Xalapa, Veracruz (Mexico), where they were interviewed. Data used to identify and locate the patients were obtained from the unit's epidemiological surveillance records, which documented the use of diagnostic tests. The inclusion criteria comprised children and adolescents under 18 years of age with symptomatic COVID-19 confirmed by rapid antigen testing or polymerase chain reaction (PCR), whose parents participated as respondents.

Two distinct manifestations of long COVID were considered: (a) “persistence”, defined as continuous symptoms beginning in the acute phase and lasting for more than 3 months; and (b) “post-COVID conditions”, defined as new or recurrent symptoms lasting for more than 3 months, appearing after the acute episode, and not associated with any active disease or infectious condition. Regarding the latter criterion, a complete medical evaluation of the child was carried out by a trained physician to detect infectious conditions or diseases causing the reported symptoms; if necessary, complementary laboratory or imaging tests were performed.

The occurrence of a subsequent acute episode was evaluated using PCR, with the results obtained retrospectively from the epidemiological surveillance records; only cases with a positive result were considered to be SARS-CoV-2 reinfections, in which case the 3-month evaluation period was restarted.

**Figure 1:**
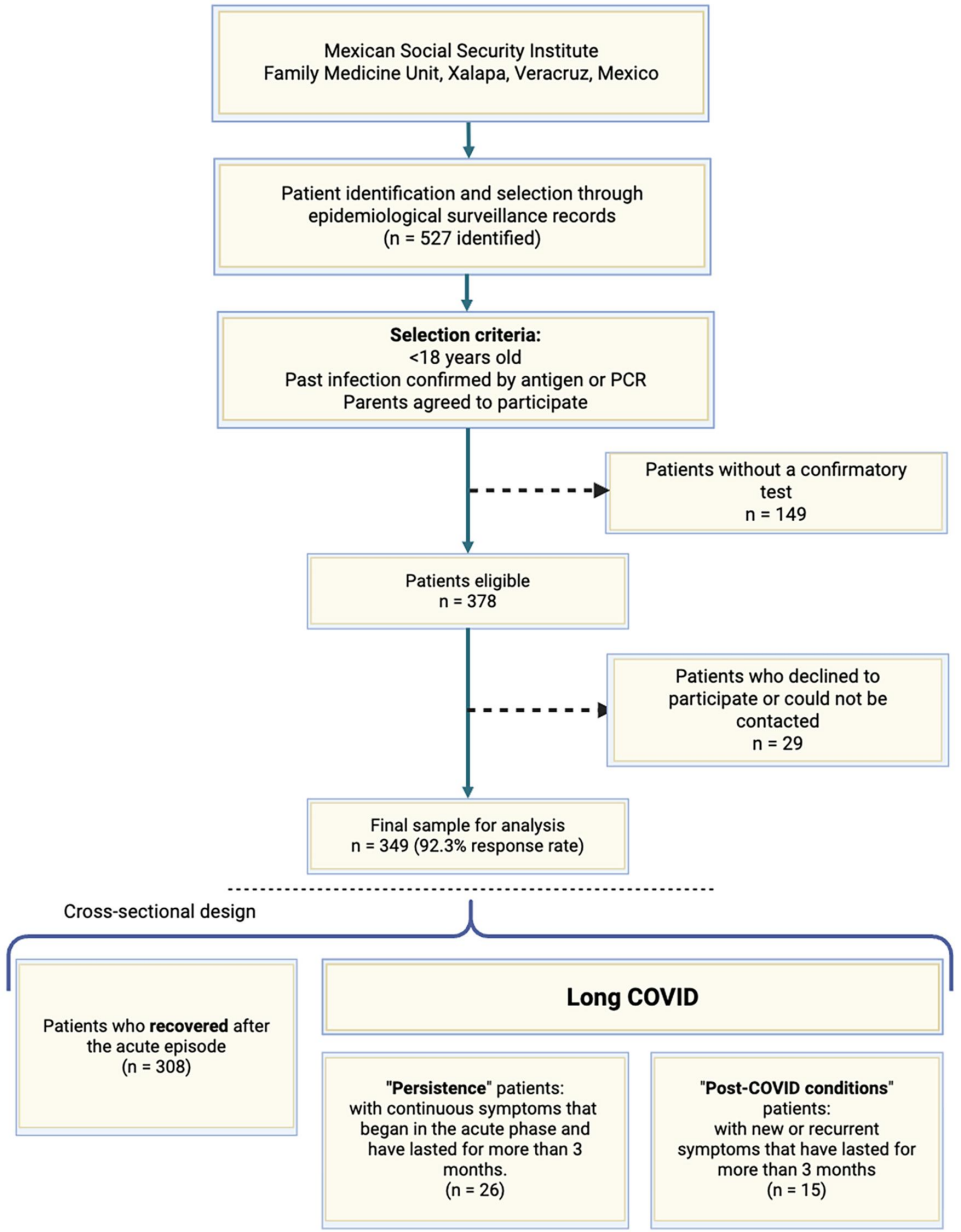
General flowchart of the study design. Created in BioRender, available at: https://BioRender.com/vfxjoav

The exclusion criteria included cognitive, intellectual, or hearing impairments in parents, preventing completion of the questionnaire. At the time of the study, COVID-19 vaccination had not yet been approved for children in Mexico; therefore, none of the participants had been vaccinated (the vaccination period for children over 12 years old began at the end of 2022 in Mexico). Furthermore, the study population corresponded to a primary outpatient care setting, as defined by national healthcare protocols during the health emergency; patients with severe disease were referred to specialized hospitals. All patients who met the selection criteria and whose parents provided informed consent were included. This process is illustrated in Figure 1.

### 2.2 Data collection and processing

A structured questionnaire was used to record the following: age at COVID-19 diagnosis (acute phase); sex; weight, height, and body mass index (BMI). In addition, a clinical interview was conducted to identify signs and symptoms associated with long COVID in children, including their presence and duration. The symptoms assessed included fever, myalgia, arthralgia, headache, cough, anosmia, ageusia, rhinorrhea, dyspnea or shortness of breath, diarrhea, fatigue, sore throat, hyporexia (loss of appetite), asthenia, vertigo/dizziness, constipation, and parental perceptions of irritability, confusion, memory loss, anxiety, and depression. Family type (20) and Geyman's family life cycle phase (21) were also recorded.

Clinical interviews were the primary method used to identify symptoms and were conducted by a primary care physician, who inquired about the presence of the listed symptoms. The selection of the investigated signs and symptoms was guided by parent-reported instruments, following the methodology proposed by Asadi-Pooya et al. (22). In addition, medical records were reviewed, and for children aged over 8 years, the child was also interviewed to supplement and confirm parental reports.

A descriptive analysis was performed using summary statistics and measures of central tendency. For the comparison of categorical variables, the chi-square test with Mantel–Haenszel correction or Fisher's exact test was used; for continuous variables, a Student's t-test or the Mann–Whitney *U*-test was employed. An exploratory block binary logistic regression analysis (enter method) was used to identify associated factors through odds ratios (ORs), with the presence or absence of long COVID (including both “persistence” and “post-COVID conditions”) considered the dependent variable in all models.

The independent variables were entered as a block in the following order: SARS-CoV-2 reinfection, time elapsed since the acute episode, BMI, sex, and finally, age. Based on these results, significant, borderline significant, and potentially confounding variables were used to construct four multivariable models in the study sample, which was stratified into two groups according to patient age: ≥ 8 years and < 8 years. This stratification was applied because children aged ≥ 8 years were able to respond to questions about their illness, whereas children aged < 8 years relied solely on parental reports. Model I included only the variable “SARS-CoV-2 reinfection” (unadjusted); additional variables were introduced sequentially until the final Model IV (fully adjusted) was obtained. Independent variables demonstrating collinearity, defined as a variance inflation factor (VIF) > 5, were excluded. Model fit was assessed using the Hosmer–Lemeshow test (*p* > 0.05). The MID-P method was used to calculate confidence intervals for prevalence estimates. All analyses were performed using IBM SPSS Statistics for Mac, version 29.0.

### 2.3 Ethical approval

The study protocol was approved by the Research Committee and the Research Ethics Committee of the Mexican Social Security Institute (registration number R-2023-3007-047). All parents were informed about the purpose of the study and provided informed consent. In addition, all children aged over 8 years were asked to provide assent, as their parents were supplying personal information on their behalf.

## 3 Results

### 3.1 Population characteristics

The study included 349 children and adolescents (response rate: 92.3%). The general characteristics of the study population are summarized in Table 1. Age at diagnosis ranged from 10 months to 18 years, with a median of 12 years [interquartile range (IQR): 6 years]. The male sex, at 51.9% of the sample, comprised the majority of participants. Regarding family characteristics, the majority of children belonged to simple nuclear families (80.5%); 98.9% of the families resided in urban areas, 66.5% were classified as modern families, and 64.2% were engaged in the services sector. The above refers to the father with the greatest share in the family income.

**Table 1:** General characteristics of the study population (*n* = 349).

Variable	Summary Statistic	Dispersion
Men	181	51.9
Women	168	48.1
Age group at the time of acute COVID-19 infection *(n, %)*		
Less than 2 years old	15	4.3
2–5 years old	22	6.3
Over 5 years old	312	89.4
Body mass index at the time of the survey by age group *(P_50_, P_75 −_ P_25_)*		
>1 year old	21.5	19.2,29
1 year old	16.1	14.6,20.1
2–4 years old	16.5	15.6,18
5–9 years old	18.6	16.2,21.5
10–14 years old	21.5	18.5,23.6
15–18 years old	23.3	21.5,25.6
Body mass index at the time of the survey (overall) *(P_50_, P_75 −_ P_25_)*	21.4	17.9,23.8
Time elapsed since the acute episode *(n, %)*		
3–6 months	4	1.2
>6–12 months	95	27.2
>12–24 months	246	70.5
>24 months	4	1.1
Family classification based on kinship *(n, %)*		
Simple nuclear	281	80.5
Numerous nuclear	14	4.0
Reconstructed	1	0.3
Single-parent	51	14.6
Extended single-parent	1	0.3
Extensive compound	1	0.3
Family classification based on physical presence in the home *(n, %)*		
Integrated core	280	80.2
Non-integrated core	68	19.5
Extensive ascending	1	0.3
Family classification based on its demographics *(n, %)*		
Urban	345	98.9
Rural	4	1.1
Family classification based on its development *(n, %)*		
Traditional	117	33.5
Modern	232	66.5
Family classification based on occupation *(n, %)*		
Industrial	34	9.7
Commercial	91	26.1
Services	224	64.2
Classified BMI^a^ at the time of the survey		
Low weight	12	3.4
Suitable weight	214	61.3
Overweight	67	19.2
Obesity	56	16.1
Presence or absence of long COVID *(n, %)*		
Presence of long COVID	41	11.8
Absence of long COVID	308	88.3
Presence or absence of long COVID (subclassified into “post-COVID conditions” and “persistence” groups) *(n, %)*		
No long COVID	308	88.3
Long COVID (post-COVID conditions)	15	4.4
Long COVID (Persistence)	26	7.5
Long COVID (Persistence and/or post-COVID conditions)	41	11.8
SARS-CoV-2 reinfection *(n, %)*		
No	340	97.4
Yes	9	2.6
Weight at the time of the survey (Kilograms) *(*P_50_, P_25_ - P_75_*)*	48	31,60
Height at the time of the survey (Meters) (P_50_, P_25_ - P_75_)	1.5	1.3,1.6
Age at the time of acute COVID-19 (P_50_, P_25_ - P_75_)	12	9,15
Number of signs or symptoms in those in the persistence group (P_50_, P_25_ - P_75_)	1	1,1
Number of signs or symptoms in those in the post-COVID conditions group (P_50_, P_25_ - P_75_)	1	1,2

NC not calculable, P_50_: median, P_25_: 25th percentile, P_75_: 75th percentile. BMI: Body mass index (kg/m^2^). NA: Not applicable.

a To classify BMI at the time of the survey, the WHO z-score was used according to the following divisions: for children under 2 years of age, weight-for-length was used; for children aged 2–5 years, weight-for-height was used; and for children over 5 years, BMI tables were used.

### 3.2 Prevalence and symptoms of long COVID

**Figure 2:**
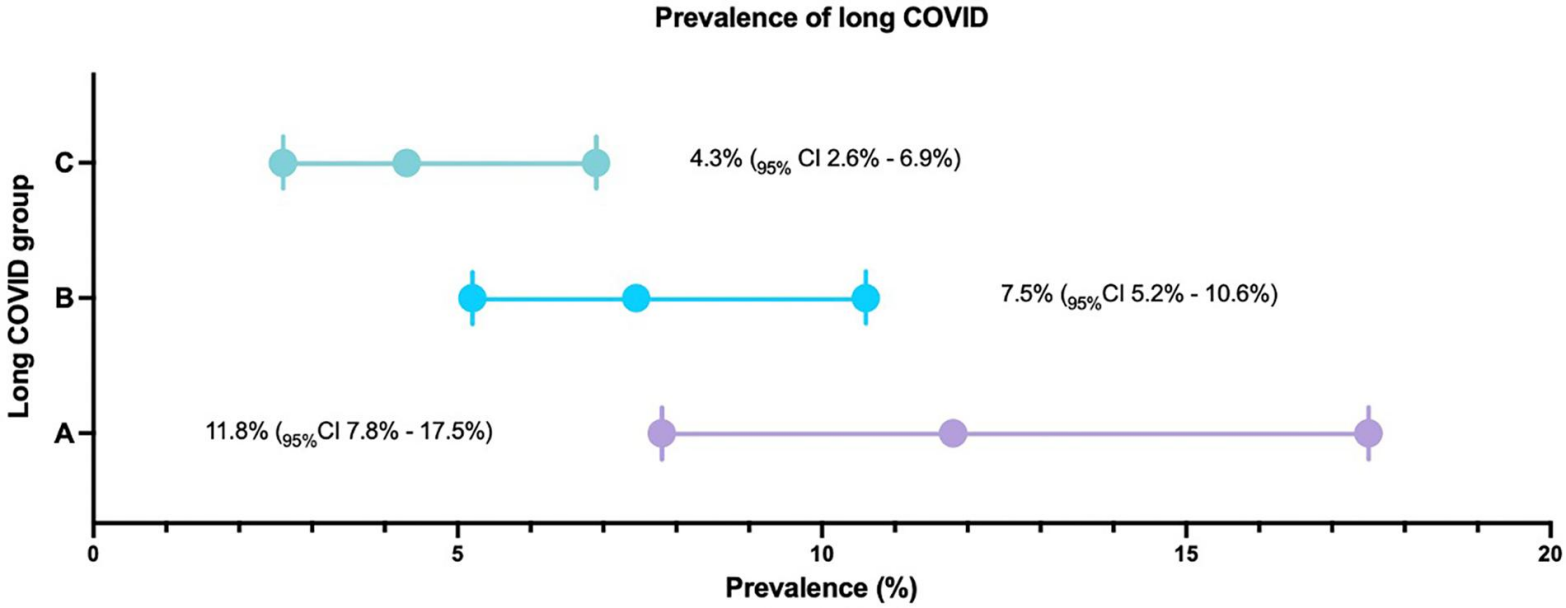
Prevalence of long COVID in children and adolescents. A) Long COVID (Persistence or/and post-COVID conditions groups included). B) Long COVID (persistence group only). C) Long COVID (post-COVID conditions group only). Created in GraphPad Prism 11.0.2.

The prevalence of long COVID was estimated at 11.8% [95% confidence interval (_95%_CI): 7.8%–17.5%], with 7.5% (_95%_CI: 5.2%–10.6%) corresponding to “persistence” and 4.3% (_95%_CI: 2.6%–6.9%) to “post-COVID conditions” (Figure 2). A total of 2.5% (9 cases) had a history of reinfection following the acute episode. Symptoms observed during the acute phase and in long COVID cases are presented in Tables 2, 3, respectively. The most prevalent symptoms during the acute phase were fever (69.3%), headache (69.6%), rhinorrhea (64.5%), cough (63.3%), odynophagia (55.6%), and myalgia (50.7%). In “persistence” cases, the most frequent symptoms were cough (50%) and rhinorrhea (15.4%). In contrast, in “post-COVID conditions”, the most common symptoms were myalgia (33.3%), asthenia and irritability (26.7% each), and constipation (20%).

**Table 2:** Prevalence of signs and symptoms in subjects with acute COVID-19.

Symptom	Acute COVID-19 *n* = 349	
	n	%
Headache		
No	106	30.4
Yes	243	69.6
Fever		
No	107	30.7
Yes	242	69.3
Rhinorrhea		
No	124	35.5
Yes	225	64.5
Cough		
No	128	36.7
Yes	221	63.3
Sore throat		
No	155	44.4
Yes	194	55.6
Myalgia		
No	172	49.3
Yes	177	50.7
Arthralgia		
No	181	51.9
Yes	168	48.1
Asthenia		
No	226	64.8
Yes	123	35.2
Fatigue		
No	234	67
Yes	115	33
Anosmia		
No	249	71.4
Yes	100	28.7
Ageusia		
No	265	75.9
Yes	84	24.1

Only the 11 most relevant signs or symptoms in patients with acute COVID-19 were included; the complete table can be found in the Supplementary Material.

**Table 3:** Prevalence of signs and symptoms in long COVID (divided into “persistence” and “post-COVID conditions” groups).

Symptom	Long COVID			
	Persistence		Post-COVID conditions	
	(*n* = 26)	%	(*n* = 15)	%
Arthralgia				
No	26	100.00	13	86.7
Yes	0	0.00	2	13.3
Myalgia				
No	26	100.00	10	66.7
Yes	0	0.00	5	33.3
Dyspnea or shortness of breath				
No	25	96.1	15	100.0
Yes	1	3.9	0	0.0
Cough				
No	13	50.0	13	86.7
Yes	13	50.0	2	13.3
Diarrhea				
No	24	92.3	13	86.7
Yes	2	7.7	2	13.3
Ageusia				
No	24	92.3	14	93.3
Yes	2	7.7	1	6.7
Rhinorrhea				
No	22	84.6	14	93.3
Yes	4	15.4	1	6.7
Asthenia				
No	25	96.1	11	73.3
Yes	1	3.9	4	26.7
Irritability				
No	24	92.3	11	73.3
Yes	2	7.7	4	26.7
Anxiety				
No	25	96.1	13	86.7
Yes	1	3.9	2	13.3

Only the 10 most relevant signs or symptoms in patients in the persistence or post-COVID conditions groups were included; the complete table can be found in the Supplementary Material.

### 3.3 Factors associated with long COVID

Table 4 presents factors associated with long COVID that increased its probability, including male sex and a shorter time since the acute episode (< 1 year). Factors associated with a reduced probability included older age at the time of acute illness, a higher BMI at the time of the survey, and more time elapsed since the acute episode. All these associations had *p*-values < 0.05. No variables were significantly associated with the subdivision between “persistence” and “post-COVID conditions” (see Supplementary Material Table S4).

**Table 4:** Univariate analysis of factors associated with long COVID in children and adolescents.

Variables	Categories	Without long COVID (*n* = 308)		With long COVID (*n* = 41)		*p*-value	*^OR (CI)^95%* *(Not adjusted)*
		n	%	n	%		
Sex	Male	153	49.7	28	68.3	0.03ª	2.2* (1.1–4.4)
	Female	155	50.3	13	31.7		
SARS-CoV-2 reinfection	No	302	98.1	38	92.7	0.07^b^	3.9 (0.95–16.5)
	Yes	6	2.0	3	7.3		
Time elapsed since the acute episode	< 1 year	73	23.7	26	63.4	<0.01^a^*	5.6* (2.8–11.1)
	1 year or more	235	76.3	15	36.6		
Classified BMI^e^ (at the time of the survey)	Adequate weight	193	62.7	21	51.2	0.16^c^	1.6 (0.8–3.1)
	Underweight or overweight	115	37.3	20	48.8		
Variables		P_50_	p25,p75	P50	p25,p75	*p*-value	*^OR (CI)^95% (Not adjusted)*
Age at acute illness (older age)		13	10,16	7	1.8,11	<0.01^d^*	0.78* (0.72–0.84)
BMI at the time of the survey (higher)		21.6	18.3,24	18.3	16.4,21.5	<0.01^d^*	0.86* (0.78–0.94)
Time elapsed since the acute episode (in months)		451	384,555	313	252, 437	<0.01^d^*	0.79* (0.73–0.87)

Statistical tests used: ^a^Pearson Chi-square, ^b^Fisher Exact Test, ^c^Mantel-Haenzel correction for Chi-square, ^d^U Mann–Whitney test.

NC not calculable, Me median, P25: 25th percentile, P50: 50th percentile, P75: 75th percentile. BMI: Body mass index (kg/m^2^). NA: Not applicable.

e To classify BMI, the WHO z-score was used according to the following divisions: for children under 2 years of age, weight-for-length was used; for children aged 2 to 5 years, weight-forheight was used; and for children over 5 years, BMI tables were used.

* Statistical significance, *p*-value <0.05

Two binary logistic regression models were constructed according to patient age (≥ 8 years and < 8 years), based on the child's ability to contribute to symptom reporting. In children aged ≥ 8 years, the associated factors included a history of reinfection (OR 9.7, _95%_CI: 1.6–58) and BMI at the time of the survey (OR 1.1, _95%_CI: 1.0–1.2). In children aged < 8 years, the associated factor was male sex (OR 4.7, _95%_CI: 1.3–17.3). The full set of models is presented in Table 5.

**Table 5:** Exploratory multivariate analysis of factors associated in children with long COVID, defined as persistence (continuous) and post-COVID conditions (new or recurrent).

Variable	Model I		Model II		Model III		Modelo IV	
	OR (IC95%)	p	OR (IC95%)	p	OR (IC95%)	p	OR (IC95%)	p
Children aged ≥ 8 years (*n* = 281)								
SARS-CoV-2 reinfection	6.9 (1.2–38.6)	0.028	8.7 (1.5–52.1)	0.017	9.2 (1.5–54.7)	0.015	9.7 (1.6–58.0)	0.013
Time elapsed since the acute episode (in months)			0.8 (0.7–0.9)	0.001	0.8 (0.7–0.9)	<0.001	0.8 (0.6–1.0)	0.053
BMI (Kg/m^2^)					1.1 (0.95 to 1.2)	0.278	1.1 (1.0–1.2)	0.249
Sex (men)							0.7 (0.3–2.1)	0.572
Age (years)							0.9 (0.7–1.3)	0.648
Children aged < 8 years (*n* = 68)								
SARS-CoV-2 reinfection	1.9 (0.1–31.3)	0.663	1.7 (0.1–30.0)	0.705	2.0 (0.1–36.4)	0.636	2.0 (0.1–43.2)	0.658
Time elapsed since the acute episode (in months)			1.0 (0.9–1.2)	0.723	1.04 (0.9–1.2)	0.866	1.0 (0.8–1.2)	0.946
BMI (Kg/m^2^)					0.9 (0.7–1.0)	0.134	0.9 (0.7–1.1)	0.184
Sex (men)							4.7 (1.3–17.3)	0.020
Age (years)							0.9 (0.7–1.1)	0.340

Exploratory multivariable logistic regression models.

Time elapsed from the acute phase of COVID-19 to the day of the interview.

BMI, Body mass index at the time of the survey.

Age at diagnosis of COVID-19.

Number of reinfections per group: 2 in children under 8 years old and 7 in children over 8 years old.

OR, Odds ratio obtained through binary logistic regression.

Variables introduced using the “Enter”method.

## 4 Discussion

In Mexico, there is limited data on the prevalence of long COVID in children and adolescents, as the National Health and Nutrition Survey (ENSANUT), responsible for generating official national statistics, has focused primarily on adults (18, 23). The study with the largest pediatric population in Mexico is that described by Martínez-Valdez et al. *(*19); however, it was limited to describing the acute phase of the disease, without identifying patients who continued to experience symptoms or sequelae.

One of the most significant challenges is the definitive diagnosis of long COVID. Despite the existence of an established definition (7), the possibility that symptoms are attributable to other diseases or are coincidental continues to obscure accurate case identification. One proposed method to improve diagnostic accuracy is the establishment of a clinical “threshold” based on data from the RECOVER Pediatric Observational Cohort Study (RECOVER-Pediatrics) (17, 24). This threshold facilitates clearer case identification; that study has reported that the prevalence of at least one persistent symptom may increase long COVID prevalence to 45% in children (ages 5–12) and 39% in adolescents (ages 12–18) with a positive test result (17). When applying this threshold, the prevalence rates decrease to 20% in children and 4% in adolescents (17). These findings align more closely with those of the present study, which identified a prevalence of 11.8% (_95%_CI 7.8%–17.5%) even without applying a threshold, with higher prevalence rates observed in younger patients at diagnosis. This consistency supports the plausibility of our findings.

A key finding of the present study was the prevalence of 11.8% (_95%_CI 7.8%–17.5%) in children and adolescents without prior vaccination following a symptomatic acute episode. Comparison with other studies is challenging due to differences in selection criteria. When standardizing for symptomatic cases with at least 3 months of follow-up, however, our findings can be compared with the 4.8% prevalence reported in the German study “COVID-19/SARS-CoV-2 seroconversion in children” (CorKid) (10, 25), which focused on the Omicron variant. For earlier variants, a prevalence of 14.2% has been reported (10, 25). It is important to note that the comparative study had a smaller sample size than ours. Cough and dyspnea were frequent respiratory symptoms, consistent with our findings; cough was more prevalent in the “persistence” group than in the “post-COVID conditions” group (50% vs. 13.3%). Differences in prevalence may be explained by variations in follow-up duration, severity of acute illness, age, and SARS-CoV-2 variants. Unfortunately, stratification by viral strain was not possible in our study, as only a small proportion of PCR-positive cases were subtyped in Mexico.

Another relevant finding of the present study was the association between male sex and increased probability of long COVID in children under 8 years of age (OR 4.7, _95%_CI: 1.3–17.3). This is consistent with findings in adult populations, where this effect has been linked to hormonal, immunological, and receptor expression factors (26).

A systematic review of 349 children and adolescents reported a global prevalence of long COVID of 25.24%, with the most frequent symptoms including mood disturbances (sadness, tension, anger, depression, anxiety) in 16.5% of the subjects, fatigue in 9.62% of the subjects, sleep disorders in 8.42% of the subjects, headaches in 7.84% of the subjects, and respiratory symptoms in 7.62% of the subjects. Comparison was complicated by variation in follow-up duration, which ranges from 4 to 12 weeks or longer (27). This may explain the lower prevalence observed in our study. These variations highlight the importance of studying different populations and contexts.

The instrument proposed by Asadi-Pooya et al. *(*22) is among the simplest and most effective tools for identifying long COVID in pediatric populations. Using this instrument, a prevalence of 44.8% was reported in that study, with the most frequent symptoms being fatigue (21%), shortness of breath (12%), cough (7%), muscle and joint pain (5%), and headache (5%). These findings differ from ours, where objective symptoms such as cough and rhinorrhea were more prevalent. This discrepancy may be explained by reliance on parent-reported data. Although the data collection methods were similar, our study incorporated both parental and child reports where possible, depending on the child's age.

Regarding symptom prevalence, the review by Pellegrino et al. (8) identified fatigue, headache, muscle and joint pain, chest pain, dyspnea, and alterations in the sense of taste or smell as the most frequent symptoms. In our study, only headache was identified as a high-frequency symptom. Similarly, Basaca et al. (9) reported headache prevalence ranging from 3% to 80%, along with dizziness and memory loss, both of which were relatively uncommon in our sample (3.9%–6.7%).

A systematic review in otorhinolaryngology (12) identified cough as a frequent symptom (4%–19%), along with anosmia (14% at 60 days) and dysgeusia (12% at 60 days), both of which were more common than in our findings. It is noteworthy that symptoms tend to decrease over time, a pattern also observed in our study: one year was sufficient for a significant reduction in long COVID cases.

Vaccination has been shown to not only reduce the incidence of acute symptomatic disease and severe forms but also to decrease the likelihood of developing long COVID (28). However, due to delays in vaccine availability, none of the study participants had been vaccinated at the time of the study (29).

The long-term effects of long COVID in children and adolescents remain uncertain. These may range from measurable outcomes, such as increased mortality (30), to broader impacts such as rising healthcare costs (16), reduced physical activity in children and adolescents (15), and widening social and economic inequalities (31). This underscores the need for continued research, improved diagnostic tools, and the development of targeted care programs.

Regression analysis of children aged ≥ 8 years identified reinfection as a factor associated with an increased probability of long COVID (OR 9.7, _95%_CI: 1.6–58). This is consistent with findings in adult populations, where the risk of long COVID increases with each reinfection (32), particularly when the intervals between infections are shorter (33). However, it is important to mention that the number of confirmed reinfections for each analyzed group is small: 7 for the group over 8 years old and 2 for the group under 8 years old, so the results should be interpreted with caution. It is necessary to continue investigating the effect of reinfection confirmed by viral identification on the prevalence of long COVID.

This study has several limitations, including its cross-sectional design, which precludes causal inference, potential recall bias, and a relatively limited sample size. Additionally, asymptomatic cases were excluded due to healthcare protocols during the pandemic, which prioritized testing for symptomatic individuals. Parent-reported data may overemphasize observable symptoms (e.g., cough) while underrecognizing subjective symptoms, such as fatigue or cognitive changes, particularly in younger children. The inclusion of children aged ≥ 8 years was therefore essential to capture subjective symptoms. The small sample size in the subgroup of children < 8 years may also have introduced a type II error. Furthermore, the small number of reinfections reduced precision, as reflected in wide confidence intervals. An important limitation is the small number of children under 2 years of age (15 children, 4.3%) and those aged 3–5 years (22 children, 6.3%). This may reflect a higher proportion of asymptomatic cases in these age groups and the difficulty parents face in recognizing symptoms, as these children rely on third-party identification and reporting. Finally, because reinfection was observed in only nine participants, the association estimates are imprecise and should not be interpreted as confirmatory.

The strengths of this study include: (1) strict selection criteria; (2) confirmation of SARS-CoV-2 infection in all participants; (3) use of a contemporary definition of long COVID (7); and (4) a high response rate, reflecting a population engaged with healthcare research.

## 5 Conclusion

Long COVID remains a relevant and complex condition that requires continued attention, including in children and adolescents. This study identified a prevalence of 11.8% (_95%_CI 7.8%–17.5%) among unvaccinated symptomatic patients treated in primary care, underscoring the need for greater clinical awareness in routine practice.

Failure to recognize long COVID may lead to misdiagnosis and suboptimal treatment. Identifying factors associated with each age group, such as sex and a history of reinfection, may help prioritize monitoring and care for vulnerable groups. It is important to remember that this article serves as a hypothesis generator that requires subsequent research to confirm the findings. Further research is needed to standardize diagnostic criteria, assess long-term outcomes, and develop evidence-based treatment strategies in pediatric populations, particularly in settings with limited existing data, such as Mexico.

## Data Availability

The raw data supporting the conclusions of this article will be made available by the authors, without undue reservation.
