# Supplementary material for: SARS-CoV-2 reinfection: a possible contributing factor to long COVID in children and adolescents
Source: Front Pediatr. 2026 Jun 16;14:1691052. doi: 10.3389/fped.2026.1691052 (PMC13314616; doi:10.3389/fped.2026.1691052)
Supplement: Supplementary file 1 [file Table1.docx]

| Index  Table S1. *Descriptive statistics of continuous study variables.*  Table S2. *Prevalence of signs and symptoms in acute and Long-Covid (divided into persistence and post-COVID conditions)*  Table S3. *Table S3. Descriptive statistics of continuous study variables, classified according to those without or with Long-COVID (persistence + post-COVID conditions))*  Table S4 Table S4. Differences between children and adolescents according to the characteristics of Long-COVID: persistence or post-COVID conditions  To choose the best statistic for the description of the variables, normality test was performed, *table S1* shows these values.  For normality, the Kolmogorov-Smirnov test was used wen the subsample size was greater than 50, and Shapiro-Wilks when it was not. The median was used as a comparison statistic due to the lack of normality assumption in continuous data.   \| *Table S1. Descriptive statistics of continuous study variables.* \| \| \| \| \| \| \| \| \| \| \| --- \| --- \| --- \| --- \| --- \| --- \| --- \| --- \| --- \| --- \| \| Variable \| x \| P_50_ \| s \| d \| Mi \| Mx \| R \| IQR \| *Normality*  *p-value* \| \| Weight at the time of the survey (Kilograms) \| 46.8 \| 48 \| 417.1 \| 20.4 \| 6.5 \| 112 \| 105.5 \| 29 \| 0.01^a^* \| \| Height at the time of the survey (Meters) \| 1.4 \| 1.5 \| 0.07 \| 0.3 \| 0.6 \| 1.9 \| 1.4 \| 0.3 \| <0.01^a^* \| \| Age at the time of the survey \| 13.2 \| 13.7 \| 23.4 \| 4.8 \| 0.4 \| 20.5 \| 20.1 \| 6.9 \| <0.01^a^* \| \| Body mass index at the time of the survey \| 21.5 \| 21.4 \| 21.6 \| 4.7 \| 12.6 \| 41.7 \| 29.1 \| 5.8 \| <0.01^a^* \| \| Age at the time of acute COVID-19 \| 11.6 \| 12 \| 20.7 \| 4.5 \| 0.1 \| 18 \| 17.9 \| 6 \| <0.01^a^* \| \| Days elapsed after acute COVID-19 \| 445.6 \| 440 \| 16648 \| 129 \| 87 \| 793 \| 706 \| 201 \| <0.01^a^* \| \| Months after acute COVID-19 \| 14.9 \| 15 \| 18.5 \| 4.3 \| 2.9 \| 26.4 \| 23.5 \| 6.7 \| <0.01^a^* \| \| Number of signs symptoms in those with long COVID (persistence) \| 1.2 \| 1 \| 0.2 \| 0.5 \| 1 \| 3 \| 2 \| 0 \| <0.01^b^* \| \| Number of signs symptoms in those with long COVID (post-COVID conditions) \| 1.5 \| 1 \| 1.2 \| 1.1 \| 1 \| 7 \| 6 \| 1 \| <0.01^b^* \| \| Statistical test used: ^a^Kolmogorov-Smirnov, ^b^Shapiro-Wilks.  Abbreviations: NC not calculable, x: Mean, P50: median, P25: 25th percentile, P75: 75th percentile, d: standard deviation, s: variance, IQR: interquartile range, Mi: Minimum, Mx: Maximum, R: range. BMI: Body mass index (kg/m2). NA: Not applicable \| \| \| \| \| \| \| \| \| \|   *Table S2* describes all the signs and symptoms present in patients during the acute phase of COVID-19 and in those that presented persistence or post-COVID conditions. Those with the greatest interest or relevance were concentrated in the main article.   \| *Table S2. Prevalence of signs and symptoms in acute and Long-Covid (divided into persistence and post-COVID conditions)* \| \| \| \| \| \| \| \| --- \| --- \| --- \| --- \| --- \| --- \| --- \| \| Sign or symptom \| Acute COVID-19 \| \| Long-COVID \| \| \| \| \| Persistence \| \| Post-COVID conditions \| \| \| (n=349) \| % \| (n=26) \| % \| (n=15) \| % \| \| Fever \| \| \| \| \| \| \| \| No \| 107 \| 30.7 \| 26 \| 100.0 \| 15 \| 100.0 \| \| Yes \| 242 \| 69.3 \| 0 \| 0.0 \| 0 \| 0.0 \| \| Arthralgia \| \| \| \| \| \| \| \| No \| 181 \| 51.9 \| 26 \| 100.0 \| 13 \| 86.7 \| \| Yes \| 168 \| 48.1 \| 0 \| 0.0 \| 2 \| 13.3 \| \| Myalgia \| \| \| \| \| \| \| \| No \| 172 \| 49.3 \| 26 \| 100.0 \| 10 \| 66.7 \| \| Yes \| 177 \| 50.7 \| 0 \| 0.0 \| 5 \| 33.3 \| \| Dyspnea or shortness of breath \| \| \| \| \| \| \| \| No \| 329 \| 94.3 \| 25 \| 96.2 \| 15 \| 100.0 \| \| Yes \| 20 \| 5.7 \| 1 \| 3.8 \| 0 \| 0.0 \| \| Headache \| \| \| \| \| \| \| \| No \| 106 \| 30.4 \| 26 \| 100.0 \| 15 \| 100.0 \| \| Yes \| 243 \| 69.6 \| 0 \| 0.0 \| 0 \| 0.0 \| \| Cough \| \| \| \| \| \| \| \| No \| 128 \| 36.7 \| 13 \| 50.0 \| 13 \| 86.7 \| \| Yes \| 221 \| 63.3 \| 13 \| 50.0 \| 2 \| 13.3 \| \| Diarrhea \| \| \| \| \| \| \| \| No \| 287 \| 82.2 \| 24 \| 92.3 \| 13 \| 86.7 \| \| Yes \| 62 \| 17.8 \| 2 \| 7.7 \| 2 \| 13.3 \| \| Anosmia \| \| \| \| \| \| \| \| No \| 249 \| 71.4 \| 24 \| 92.3 \| 15 \| 100.0 \| \| Yes \| 100 \| 28.7 \| 2 \| 7.7 \| 0 \| 0.0 \| \| Ageusia \| \| \| \| \| \| \| \| No \| 265 \| 75.9 \| 24 \| 92.3 \| 14 \| 93.3 \| \| Yes \| 84 \| 24.1 \| 2 \| 7.7 \| 1 \| 6.7 \| \| Sore throat \| \| \| \| \| \| \| \| No \| 155 \| 44.4 \| 25 \| 96.2 \| 15 \| 100.0 \| \| Yes \| 194 \| 55.6 \| 1 \| 3.8 \| 0 \| 0.0 \| \| Rhinorrhea \| \| \| \| \| \| \| \| No \| 124 \| 35.5 \| 22 \| 84.6 \| 14 \| 93.3 \| \| Yes \| 225 \| 64.5 \| 4 \| 15.4 \| 1 \| 6.7 \| \| Hyporexia (loss of appetite) \| \| \| \| \| \| \| \| No \| 298 \| 85.4 \| 25 \| 96.2 \| 14 \| 93.3 \| \| Yes \| 51 \| 14.6 \| 1 \| 3.8 \| 1 \| 6.7 \| \| Asthenia \| \| \| \| \| \| \| \| No \| 226 \| 64.7 \| 25 \| 96.2 \| 11 \| 73.3 \| \| Yes \| 123 \| 35.3 \| 1 \| 3.8 \| 4 \| 26.7 \| \| Irritability \| \| \| \| \| \| \| \| No \| 325 \| 93.1 \| 24 \| 92.3 \| 11 \| 73.3 \| \| Yes \| 24 \| 6.9 \| 2 \| 7.7 \| 4 \| 26.7 \| \| Anxiety \| \| \| \| \| \| \| \| No \| 344 \| 98.6 \| 25 \| 96.1 \| 13 \| 86.7 \| \| Yes \| 5 \| 1.4 \| 1 \| 3.9 \| 2 \| 13.3 \| \| Depression \| \| \| \| \| \| \| \| No \| 348 \| 99.7 \| 26 \| 100.0 \| 14 \| 93.3 \| \| Yes \| 1 \| 0.3 \| 0 \| 0.0 \| 1 \| 6.7 \| \| Fatigue \| \| \| \| \| \| \| \| No \| 234 \| 67 \| 26 \| 100.0 \| 15 \| 100.0 \| \| Yes \| 115 \| 33 \| 0 \| 0.0 \| 0 \| 0.0 \| \| Confusion \| \| \| \| \| \| \| \| No \| 348 \| 99.7 \| 26 \| 100.0 \| 14 \| 93.3 \| \| Yes \| 1 \| 0.3 \| 0 \| 0.0 \| 1 \| 6.7 \| \| Memory loss \| \| \| \| \| \| \| \| No \| 349 \| 100.0 \| 26 \| 100.0 \| 14 \| 93.3 \| \| Yes \| 0 \| 0.0 \| 0 \| 0.0 \| 1 \| 6.7 \| \| Dizziness \| \| \| \| \| \| \| \| No \| 327 \| 93.7 \| 26 \| 100.0 \| 14 \| 93.3 \| \| Yes \| 22 \| 6.3 \| 0 \| 0.0 \| 1 \| 6.7 \| \| Constipation \| \| \| \| \| \| \| \| No \| 339 \| 97.1 \| 25 \| 96.1 \| 12 \| 80.0 \| \| Yes \| 10 \| 2.9 \| 1 \| 3.9 \| 3 \| 20.0 \|   As mentioned above, to choose the best statistic for the description of the variables, normality test was performed, *table S3* shows these values, separating those who suffer from Long-COVID or not.  For normality, the Kolmogorov-Smirnov test was used wen the subsample size was greater than 50, and Shapiro-Wilks when it was not. The median was used as a comparison statistic due to the lack of normality assumption in continuous data.   \| *Table S3. Descriptive statistics of continuous study variables, classified according to those without or with Long-COVID (persistence + post-COVID conditions)* \| \| \| \| \| \| \| \| \| \| \| \| --- \| --- \| --- \| --- \| --- \| --- \| --- \| --- \| --- \| --- \| --- \| \| Variable \| Long-COVID \| x \| P_50_ \| s \| d \| Mi \| Mx \| R \| IQR \| *Normality*  *p-value* \| \| Weight at the time of the survey (Kg) \| No \| 48.9 \| 50 \| 388 \| 20 \| 6.5 \| 112 \| 106 \| 27 \| 0.07^a^ \| \| Yes \| 31.0 \| 24 \| 364 \| 19 \| 10 \| 77 \| 67 \| 34 \| <0.01^b^* \| \| Height at the time of the survey (mts) \| No \| 1.46 \| 1.5 \| 0.06 \| 0.3 \| 0.6 \| 1.9 \| 1.37 \| 0.3 \| <0.01^a^* \| \| Yes \| 1.2 \| 1.1 \| 0.09 \| 0.3 \| 0.8 \| 1.7 \| 0.9 \| 0.6 \| <0.01^b^* \| \| Age at the time of the survey \| No \| 13.8 \| 14 \| 19.9 \| 4.5 \| 0.4 \| 20 \| 20 \| 6.5 \| 0.04^a^* \| \| Yes \| 8.1 \| 8.3 \| 20.6 \| 4.5 \| 1.7 \| 16 \| 14.3 \| 9.2 \| <0.01^b^* \| \| Body mass index at the time of the survey \| No \| 21.8 \| 21.6 \| 21.9 \| 4.7 \| 12.6 \| 41.7 \| 29.1 \| 5.7 \| <0.01^a^* \| \| Yes \| 19.2 \| 18.3 \| 15.2 \| 3.9 \| 12.6 \| 30 \| 17.4 \| 5.2 \| 0.06^b^* \| \| Age at the time of acute COVID-19 \| No \| 12.2 \| 13 \| 17.6 \| 4.2 \| 0.1 \| 18 \| 17.9 \| 6 \| 0.01^a^* \| \| Yes \| 6.9 \| 7 \| 19.3 \| 4.4 \| 0.9 \| 15 \| 14.1 \| 8.3 \| <0.01^b^* \| \| Days elapsed after acute COVID-19 \| No \| 458.7 \| 451 \| 15462 \| 124 \| 143 \| 793 \| 650 \| 172 \| 0.02^a^* \| \| Yes \| 347 \| 313 \| 14925 \| 122 \| 87 \| 649 \| 562 \| 185 \| <0.01^b^* \| \| Months after acute COVID-19 \| No \| 15.3 \| 15 \| 17.2 \| 4.1 \| 4.8 \| 26.4 \| 21.7 \| 5.7 \| 0.01^a^* \| \| Yes \| 11.6 \| 10.4 \| 16.6 \| 4.1 \| 2.9 \| 21.6 \| 18.7 \| 6.2 \| <0.01^b^* \| \| Statistical test used: ^a^Kolmogorov-Smirnov, ^b^Shapiro-Wilks.  Abbreviations: NC not calculable, x: Mean, P50: median, P25: 25th percentile, P75: 75th percentile, d: standard deviation, s: variance, IQR: interquartile range, Mi: Minimum, Mx: Maximum, R: range. BMI: Body mass index (kg/m2). NA: Not applicable \| \| \| \| \| \| \| \| \| \| \|   Finally, Table S4 shows the differences between those with persistence or post-COVID conditions. it was not included in the main article because no variable presented a statistically significant difference |  |  |
| --- | --- | --- | --- | --- | --- | --- | --- | --- | --- | --- | --- | --- | --- | --- | --- | --- | --- | --- | --- | --- | --- | --- | --- | --- | --- | --- | --- | --- | --- | --- | --- | --- | --- | --- | --- | --- | --- | --- | --- | --- | --- | --- | --- | --- | --- | --- | --- | --- | --- | --- | --- | --- | --- | --- | --- | --- | --- | --- | --- | --- | --- | --- | --- | --- | --- | --- | --- | --- | --- | --- | --- | --- | --- | --- | --- | --- | --- | --- | --- | --- | --- | --- | --- | --- | --- | --- | --- | --- | --- | --- | --- | --- | --- | --- | --- | --- | --- | --- | --- | --- | --- | --- | --- | --- | --- | --- | --- | --- | --- | --- | --- | --- | --- | --- | --- | --- | --- | --- | --- | --- | --- | --- | --- | --- | --- | --- | --- | --- | --- | --- | --- | --- | --- | --- | --- | --- | --- | --- | --- | --- | --- | --- | --- | --- | --- | --- | --- | --- | --- | --- | --- | --- | --- | --- | --- | --- | --- | --- | --- | --- | --- | --- | --- | --- | --- | --- | --- | --- | --- | --- | --- | --- | --- | --- | --- | --- | --- | --- | --- | --- | --- | --- | --- | --- | --- | --- | --- | --- | --- | --- | --- | --- | --- | --- | --- | --- | --- | --- | --- | --- | --- | --- | --- | --- | --- | --- | --- | --- | --- | --- | --- | --- | --- | --- | --- | --- | --- | --- | --- | --- | --- | --- | --- | --- | --- | --- | --- | --- | --- | --- | --- | --- | --- | --- | --- | --- | --- | --- | --- | --- | --- | --- | --- | --- | --- | --- | --- | --- | --- | --- | --- | --- | --- | --- | --- | --- | --- | --- | --- | --- | --- | --- | --- | --- | --- | --- | --- | --- | --- | --- | --- | --- | --- | --- | --- | --- | --- | --- | --- | --- | --- | --- | --- | --- | --- | --- | --- | --- | --- | --- | --- | --- | --- | --- | --- | --- | --- | --- | --- | --- | --- | --- | --- | --- | --- | --- | --- | --- | --- | --- | --- | --- | --- | --- | --- | --- | --- | --- | --- | --- | --- | --- | --- | --- | --- | --- | --- | --- | --- | --- | --- | --- | --- | --- | --- | --- | --- | --- | --- | --- | --- | --- | --- | --- | --- | --- | --- | --- | --- | --- | --- | --- | --- | --- | --- | --- | --- | --- | --- | --- | --- | --- | --- | --- | --- | --- | --- | --- | --- | --- | --- | --- | --- | --- | --- | --- | --- | --- | --- | --- | --- | --- | --- | --- | --- | --- | --- | --- | --- | --- | --- | --- | --- | --- | --- | --- | --- | --- | --- | --- | --- | --- | --- | --- | --- | --- | --- | --- | --- | --- | --- | --- | --- | --- | --- | --- | --- | --- | --- | --- | --- | --- | --- | --- | --- | --- | --- | --- | --- | --- | --- | --- | --- | --- | --- | --- | --- | --- | --- | --- | --- | --- | --- | --- | --- | --- | --- | --- | --- | --- | --- | --- | --- | --- | --- | --- | --- | --- | --- | --- | --- | --- | --- | --- | --- | --- | --- | --- | --- | --- | --- | --- | --- | --- | --- | --- | --- | --- | --- | --- | --- | --- | --- | --- | --- | --- | --- | --- | --- | --- | --- | --- | --- | --- | --- | --- | --- | --- | --- | --- | --- | --- | --- | --- | --- | --- | --- | --- | --- | --- | --- | --- | --- | --- | --- | --- | --- | --- | --- | --- | --- | --- | --- | --- | --- | --- | --- | --- | --- | --- | --- | --- | --- | --- | --- | --- | --- | --- | --- | --- | --- | --- | --- | --- | --- | --- | --- | --- | --- | --- | --- | --- | --- | --- | --- | --- | --- | --- | --- | --- | --- | --- | --- | --- | --- | --- | --- | --- | --- | --- | --- | --- | --- | --- | --- | --- | --- | --- | --- | --- | --- | --- | --- | --- | --- | --- | --- | --- | --- | --- | --- | --- | --- | --- | --- | --- | --- | --- | --- | --- | --- | --- | --- | --- | --- | --- | --- | --- | --- | --- | --- | --- | --- | --- | --- | --- | --- | --- | --- | --- | --- | --- | --- | --- | --- | --- | --- | --- | --- | --- | --- | --- | --- | --- | --- | --- | --- | --- | --- | --- | --- | --- | --- | --- | --- | --- | --- | --- | --- | --- | --- | --- | --- | --- | --- | --- | --- | --- | --- | --- | --- | --- | --- | --- | --- | --- | --- | --- | --- | --- | --- | --- | --- | --- | --- | --- | --- | --- | --- | --- | --- | --- | --- | --- | --- | --- | --- | --- | --- | --- | --- | --- | --- | --- | --- | --- | --- | --- | --- | --- | --- | --- | --- | --- | --- | --- | --- | --- | --- | --- | --- | --- | --- | --- | --- | --- | --- | --- | --- | --- | --- | --- | --- | --- | --- | --- | --- | --- | --- | --- | --- | --- | --- | --- | --- | --- | --- | --- | --- | --- | --- | --- | --- | --- | --- | --- | --- | --- | --- | --- | --- | --- | --- | --- | --- | --- | --- | --- | --- | --- | --- | --- | --- | --- | --- | --- | --- |

| Table S4. Differences between children and adolescents according to the characteristics of Long-COVID: persistence or post-COVID conditions | | | | | | | |
| --- | --- | --- | --- | --- | --- | --- | --- |
| Variables | Categories | Persistence  (n=26) | | Post-COVID conditions (n=15) | | p-value |  |
|  |  | n | % | n | % |  |  |
| Sex | Men | 18 | 69.2 | 10 | 66.7 | 0.56^b^ |  |
|  | Women | 8 | 30.8 | 5 | 33.3 |  |  |
| SARS-CoV-2 reinfection | No | 23 | 88.5 | 15 | 100 | 0.24^b^ |  |
|  | Yes | 3 | 11.5 | 0 | 0 |  |  |
| Time elapsed since the acute episode (months) | 3-6 months | 1 | 3.8 | 1 | 6.7 | 0.66^c^ |  |
|  | >6-12 months | 15 | 57.7 | 9 | 60 |  |  |
|  | >12-24 months | 10 | 38.5 | 5 | 33.3 |  |  |
| Time elapsed since the acute episode (years) | <1 year | 16 | 61.5 | 10 | 66.7 | 0.74^a^ |  |
|  | 1 year or more | 10 | 38.5 | 5 | 33.3 |  |  |
| Variables | | P_50_ | IQR | P_50_ | IQR | p-value |  |
| Age at acute illness | | 7 | 8.1 | 4 | 9 | 0.44^d^ |  |
| BMI at acute illness | | 18.3 | 5.6 | 18.5 | 4.9 | 0.93^d^ |  |
| Time elapsed since the acute episode (days) | | 326 | 190 | 313 | 194 | 0.66^d^ |  |
| Time elapsed since the acute episode (months) | | 17.9 | 6.3 | 10.4 | 6.5 | 0.66^d^ |  |
| Statistical test used: ^a^Pearson Chi-square, ^b^Fisher Exact Test, ^c^Mantel-Haenzel correction for Chi-square, ^d^U Mann-Whitney test.  Abbreviations: NC not calculable, x: Mean, P50: median, P25: 25th percentile, P75: 75th percentile, d: standard deviation, s: variance, IQR: interquartile range, Mi: Minimum, Mx: Maximum, R: range. BMI: Body mass index (kg/m2). NA: Not applicable | | | | | | | |
